# Dr. Elisha G. Tucker

**Published:** 1895-07

**Authors:** 


### DR. ELISHA G. TUCKER.

THE passing away of an active member of the dental profession is always of serious moment to his colleagues, as it means a vacant place and a proportionate loss to the well-being of the organization. This feeling should be peculiarly prominent when one by one the earlier workers, the fathers, as we love to call them, are passing on to their great reward.

It is with sentiments of unusual regret that we chronicle the death of Dr. Elisha G. Tucker. While long passed the age usually allotted to men, he has been continually active and most affectionately regarded by his immediate colleagues, and the profession generally, as one of the very few who represented the earnest work of the first half of the present century, a period fraught with a larger amount of development in the practical side of dentistry than any since, and in which Dr. Tucker took a most prominent and worthy part.

We are on the threshold of another century, and as the silent reaper is gathering in the ripened harvest of this, let us indulge the hope that the example of this noble life may extend into the next century, unfolding through its inspirations increase of power and fuller development in those things which made Dr. Tucker great in his generation.

Elisha G. Tucker was born in Winchendon, Massachusetts, August 18, 1808. He received the degree of M.D. at the Berkshire Medical College, Pittsfield, Massachusetts, in 1837.

In 1838 he formed a copartnership in New York with the late Joseph H. Foster, M.D., buying out the practice of Dr. Horace Kimball, of Park Place, New York.

He removed to Boston in 1841 and joined his brother, Dr. Joshua Tucker, the connection proving eminently successful. This continued for ten years, or until 1851, when he decided to pursue his profession alone.

He was a member of the American Society of Dental Surgeons, and delivered a lecture at Newport, Rhode Island, in 1846, on the use and application of rubber rings designed for irregularities of the teeth.

In 1857 he was elected Vice-President of the American Dental Convention assembled at Boston.

He was deeply interested in the American Academy of Dental Science, of Boston, and was one of the founders of that society, and was successively treasurer, chairman of the Board of Censors, and subsequently was for two years the presiding officer of the Academy. He was at a later period elected an honorary member.

He was married in 1843 to Elizabeth M. Harris, of Portsmouth, New Hampshire.

His son, Winslow Lewis Tucker, became associated with his father in 1872.

He leaves a widow and an only son.

